# Reviewer acknowledgement 2015

**DOI:** 10.1186/s40101-016-0087-5

**Published:** 2016-02-16

**Authors:** Akira Yasukouchi

**Affiliations:** Department of Physiological Anthropology, Faculty of Design, Kyushu University, Fukuoka, Japan

## Abstract

The editors of *Journal of Physiological Anthropology* would like to thank all our reviewers who have contributed to the journal in Volume 34 (2015).

**Daijiro Abe**

Japan

**Ferihan Ahmed-Popova**

Bulgaria

**Tomoko Aoki**

Japan

**Kiyoshi Aoyagi**

Japan

**Michiko Asano**

Japan

**Kurt Beil**

United States

**Alison Bested**

Canada

**Kaushik Bose**

India

**Tom Brutsaert**

United States

**Václav Bunc**

Czech Republic

**Damee Choi**

Japan

**Seong-Woo Choi**

South Korea

**Chin Mei Chou**

Taiwan

**Aitor Coca**

United States

**Alessandro Corti**

Italy

**Douglas E. Crews**

United States

**Yuki Fujita**

Japan

**Katsuo Fujiwara**

Japan

**Takako Fukazawa**

Japan

**Yoshiyuki Fukuoka**

Japan

**Anuradha Godavarty**

United States

**Elena Godina**

Russia

**Michelle Gray**

United States

**Esperanza Gutierrez**

Spain

**Nobuko Hashiguchi**

Japan

**Chew Kiat Heng**

Singapore

**Michael Hermanussen**

Germany

**Ronald Heus**

Netherlands

**Mutsuko Hieda**

Japan

**Shigekazu Higuchi**

Japan

**Yuki Hikihara**

Japan

**Kohji Hirakoba**

Japan

**Masahiro Horiuchi**

Japan

**Foo Leng Huat**

Malaysia

**Ritsuko Imamura**

Japan

**Keita Ishibashi**

Japan

**Koichi Iwanaga**

Japan

**Emmanuel John**

United States

**Maria Kaczmarek**

Poland

**Tetsuo Katsuura**

Japan

**Sung-Phil Kim**

South Korea

**Sylvia Kirchengast**

Austria

**Shingo Kitamura**

Japan

**Naoe Kiyota**

Japan

**Takeo Kiyota**

Japan

**Hiromitsu Kobayashi**

Japan

**Miroslav Kopecký**

Czech Republic

**Kentaro Kotani**

Japan

**Katsuyasu Kouda**

Japan

**Emi Koyama**

Japan

**Tomoaki Kozaki**

Japan

**Yosuke Kusano**

Japan

**Soomin Lee**

Japan

**Chooi Yeng Lee**

Malaysia

**Michael Little**

United States

**Alan Logan**

United States

**Rie Matsubara**

Japan

**Akiyoshi Matsumura**

Japan

**Igor Mekjavic**

Slovenia

**Maija Miettinen**

Finland

**Johan Molenbroek**

Netherlands

**Takeshi Morita**

Japan

**Yuki Motomura**

Japan

**Hideyuki Mukae**

Japan

**Satoshi Muraki**

Japan

**Juan Murias**

Canada

**Andrew J. Murray**

United States

**Yumiko Nagai**

Japan

**Masashi Nakamura**

Japan

**Kazuhiro Nakayama**

Japan

**Koichi Nariyama**

Japan

**Takayuki Nishimura**

Japan

**Masato Nishiwaki**

Japan

**Hiroki Noguchi**

China

**Hidenobu Ohta**

Japan

**Akira Okada**

United States

**Renata Pacholczak**

Poland

**Giuseppe Perinetti**

Italy

**Stephane Perrey**

France

**Wolfgang Potthast**

Germany

**Ronald Prineas**

United States

**Alice Mado Proverbio**

Italy

**Sergey Rudnev**

Russia

**Yee-How Say**

Malaysia

**Christiane Scheffler**

Germany

**Eva Selhub**

United States

**Kenichi Shibuya**

Japan

**Yuji Shimizu**

Japan

**Yoshihiro Shimomura**

Japan

**Won Sop Shin**

South Korea

**Gwanseob Shin**

South Korea

**Nina Smolej Narancic**

Croatia

**Yoshiaki Sone**

Japan

**Yun-Mi Song**

South Korea

**Koji Sugiyama**

Japan

**Yuji Takasaki**

Japan

**Masato Tokui**

Japan

**Yuki Tsumura**

Japan

**Yuko Tsunetsugu**

Japan

**Janina Tutkuviene**

Lithuania

**Satoru Ueno**

Japan

**Hitoshi Wakabayashi**

Japan

**Masashi Watanabe**

Japan

**Titis Wijayanto**

Indonesia

**Shuping Xiong**

South Korea

**Shiho Yamabata**

Japan

**Kosuke Yamada**

Japan

**Naoko Yamamoto**

Japan

**Kazuhiko Yamasaki**

Japan

**Yoshiki Yasukochi**

Japan

**Takeshi Yoneshiro**

Japan

**Ai Yoto**

Japan

**Susana Zeni**

Argentina

**Javad Ziaolhagh**

Iran

